# Focusing on the Basic Principles of Dialysis to Optimize Antibiotic Therapy during Renal Replacement Therapy in Critically Ill Patients

**DOI:** 10.3390/antibiotics13090864

**Published:** 2024-09-09

**Authors:** Filippo Mariano, Alberto Mella, Luigi Biancone

**Affiliations:** 1 Department of Medical Sciences, University of Turin, 10124 Torino, Italy; alberto.mella@unito.it (A.M.); luigi.biancone@unito.it (L.B.); 2 Nephrology, Dialysis and Transplantation U, Department of General and Specialized Medicine, City of Science and Health, CTO Hospital, 10126 Turin, Italy

## 1. Introduction

Bacterial infections frequently occur in patients in the ICU undergoing renal dialysis using extracorporeal procedures (KRT) that can be applied for different time periods, such as Prolonged Intermittent Renal Replacement Therapy (PIRRT) or Continuous Kidney Replacement Therapy (CKRT). Ensuring adequate antibiotic concentration is highly desirable, even if drug dosing remains challenging in this setting. In the subset of ICU patients, various factors influencing drug exposure contribute to pharmacokinetic and pharmacodynamic (PK/PD) variability, complicating the situation further. For patients receiving both intermittent (PIRRT) or continuous (CKRT) modality, this issue can be even more complex due to an additional variable. In effect, although therapeutic drug monitoring (TDM) would be the gold standard for antibiotics with a narrow therapeutic margin (e.g., aminoglycosides, glycopeptides, and polymyxins), this issue is also critical for antibiotics considered as less toxic (e.g., beta-lactams), to circumvent the risk of underexposure due to the additional extracorporeal drug clearance [1].

In severe infections, adjusting the dosage to optimize drug effectiveness through early and adequate antibiotic therapy is a key determinant of survival [2]. More data from clinical practice are urgently needed, as underexposure and overexposure to antibiotics commonly occur in patients with altered renal function who are receiving PIRRT or CKRT [3].

## 2. PK Variability of Antibiotics in ICU Patients Undergoing CKRT

It is well known that in critically ill patients with acute kidney injury (AKI) inflammation, hypoalbuminemia, modification of the cellular environment, organ failure, and tissue hypoperfusion can induce profound PK alterations affecting both the volume of distribution (Vd) and drug clearance. The Vd is usually increased as the patient presents an “inflammatory status”, an increased capillary permeability, and fluid accumulation in the interstitial compartment, often leading to an enlarged extravascular compartment. Usually, this condition in patients with severe hemodynamic instability is exacerbated by extensive intravascular fluid resuscitation. While the reduced drug clearance by renal failure can increase serum drug concentration, an enhanced Vd can result in an overall reduction in serum and tissue drug concentrations [4]. Consequently, predicting serum drug concentrations becomes challenging, especially when KRT introduces an additional variable capable of profoundly influencing the drug clearance.

In an ICU patient on KRT, the total body clearance of a drug is equal to the sum of regional clearances (renal + metabolic + other + extracorporeal). As for the various regional clearances, KRT clearance becomes clinically relevant only if its contribution to total body clearance (fractional clearance) exceeds 25%. KRT can be performed using different techniques, either in continuous (CKRT) or prolonged intermittent (PIRRT, lasting 8–12 h/day) modalities. Currently, the continuous modality (CKRT) is the most commonly used, generally carried out as hemodialysis or hemodiafiltration, with high-flux filters. These filters are equipped with membranes that are highly permeable to molecules up to 20 kDa and operate at high dialysate and low blood flow rate (usually with a blood/dialysate flow rate ratio greater than 1:3).

Therefore, the extracorporeal removal of antibiotics during CKRT primarily relies on molecule diffusion across a semipermeable membrane driven by the concentration gradient between the blood and dialysate. This process is limited by the dialysate flow rate. However, CKRT only “clears” the plasma, and antibiotics with large Vd are less efficiently removed. Only the free fraction of the antibiotic dispersed in plasma water can diffuse across the membrane [5], while the fraction bound to protein cannot. Therefore, the sieving coefficient (SC = Cdial/Cserum) of an antibiotic largely depends on its free fraction, with the SC being approximately equal to this fraction. As an additive removal mechanism, some membranes, such as PMMA and PAN, also possess an adsorption capacity of the protein-bound fraction of the molecule. However, the potential increased clearance for antibiotics with a large bound fraction is generally limited as adsorption rapidly reaches saturation [5].

From a practical point of view, in most critically ill patients undergoing CKRT, the system typically works under conditions where the dialysate flow rate is the limiting factor for removal. As a result, the filter clearance of the antibiotic is directly proportional to the product of the dialysate (filtration) rate and the drug sieving coefficient.

Cl extr = Qf × S ≈ Qf × (1 − PB)

where Cl extr = extracorporeal clearance; Qf = dialysate (filtration) rate; S = sieving coefficient; PB = protein binding; (1 − PB) = free fraction.

## 3. The Case of Colistin for Multidrug-Resistant Gram-Negative (MDRGN) Infections in Patients on CKRT

Due to the emerging resistance to carbapenems, fluoroquinolones, and aminoglycosides, the old lipopeptide antibiotic colistin (polymyxin E) has been rediscovered as an effective treatment for carbapenem-resistant *Acinetobacter baumannii*, *Pseudomonas aeruginosa*, and *Klebsiella pneumonia* infections. Colistin remains a viable alternative to newer antibiotics or combination therapies due to its lower financial cost, the occasional local unavailability of newer drugs, and because of the inconsistent clinical efficacy of those treatments against carbapenem-resistant *Acinetobacter baumannii* infections [6].

To reduce adverse effects, colistin is administered intravenously as a prodrug colistin methanesulphonate (CMS). The CMS is quickly excreted via glomerular filtration and tubular secretion, making the renal clearance of the CMS an important limiting factor in the conversion of CMS to colistin, which must occur in the plasma. In effect, patients with renal failure have more time for the generation of colistin from the prodrug CMS, as suggested by an inverse relationship between renal function and the rate and extent of CMS conversion [7].

From a PK perspective, approximately 50% of colistin binds to plasma proteins, mainly α-1-acid glycoprotein. In critically ill patients, higher levels of protein binding have been documented (59–74%), with a relatively low distribution volume (12 L) corresponding to the volume of extracellular fluid [7].

Colistin therapy remains crucial for survival in patients with severe burns who are infected with *Acinetobacter baumannii*. Recent data showed that a short course of therapy at an appropriate dosage can lead to clinical success and survival without a significant association with severe renal impairment [8].

However, the concept of an appropriate dosage of colistin is challenging in patients with septic shock-associated acute kidney injury (AKI) who require continuous venovenous hemodiafiltration (CVVHDF). The PK is affected by many variables, some intrinsic to the patient and others related to the characteristics of extracorporeal therapy (e.g., method of dialysis, filters used, potential elimination of drug amount by absorption mechanism, among others). In patients with severe renal failure, the few available PK studies have documented that the dosage of colistin should not be reduced, but rather left unchanged at the full recommended dose [9,10,11,12]. Using filters with polysulfone membranes, the mean SC values measured after 10 min of CVVHDF were 0.42 for colistin A and 0.48 for colistin B; they proved to be quite stable for 24 h, and only gradually declined to 0.24 for colistin A and 0.32 for colistin B after 48 h [9]. Additionally, the PK data indicate that extracorporeal therapy eliminated a significant portion of the drug, particularly when an absorptive component was associated with it. In patients treated with coupled plasma filtration and adsorption CKRT (CPFA-CKRT) for septic shock-associated AKI, plasma perfusion was very efficient in removing the drug, as the post-cartridge colistin concentration was not detectable [10]. Based on these PK data, to avoid the risk of colistin underdosing, patients requiring CPFA-CKRT received full doses, sometimes increased, to compensate for extracorporeal losses due to diffusion and absorption. By applying this approach of colistin administration, a CPFA benefit was observed. A significant reduction in mortality was documented in a cohort of 39 burn patients treated with CKRT-CPFA, compared to a cohort of 87 patients treated with CKRT alone (51.3% and 77.1%, respectively, *p* 0.004) [12].

The potential benefits of CPFA, first documented in the Compact study [13] and later observed in burn patients [12], were not confirmed in the Compact-2 study, which was prematurely stopped due to a suspected excess of early mortality in the CPFA-treated group [14]. The Compact-2 study compared patients with septic shock treated with CKRT- CPFA (CPFA-CKRT group) as an adjunctive therapy and patients who did not receive it (control group). Final hospital mortality was not significantly different in the CPFA-CKRT group compared to the control group (55.6% vs. 46.2%, *p* 0.35). However, an unplanned analysis showed higher mortality in the CPFA-CKRT group among patients without severe renal failure, who would not have required, per se, the renal replacement therapy (*p* = 0.025). In addition, a dose–response relationship was observed between the treated plasma volume and mortality (*p* = 0.010) [14].

In planning the trial, insufficient attention was given to the potential risk of antibiotic clearance by extracorporeal treatment. As the Authors wrote in the Discussion “…It is tempting to think that it could be harmful to subject a patient with septic shock but good renal function to RRT (even regardless of the presence of the CPFA cartridge). Doubling the purifying function could eliminate important substances at an excessive rate. The prime suspect is antibiotics, as any delay in receiving appropriate antibiotic therapy in patients with severe sepsis or septic shock is associated with excess mortality. …We calculated that CPFA removes 50% more antibiotics than standard continuous RRT, increasing the possibility of undertreatment. In this regard, a significant dose–response effect of treated plasma on mortality was demonstrated in patients without severe renal failure…” [14].

## 4. New Antibiotics in ICU Patients: Which Impact of CKRT?

The problem of MDR-resistant bacteria has become critically important in ICU patients, where the incidence and impact of these infections are major determinants of patient survival [15]. In this context, developing new agents is a problematic issue, and governance agencies (i.e., the EU COMBACTE-CARE initiative) are trying to support pharmaceutical industries in implementing this research area. For example, in April 2024, following a recommendation from the European Medicines Agency, the Commission adopted the authorization of a new antibiotic aztreonam/avibactam (Emblaveo^®^, Pfizer Europe, Bruxelles, Belgium), for complicated intra-abdominal and urinary tract infections, hospital-acquired pneumonia, and infections caused by certain types of drug-resistant bacteria [16].

To date, the primary focus has been on pathogens belonging to the so-called ESKAPE group, an acronym that stands for the *Enterococcus faecium*, *S. aureus*, *K. pneumoniae*, *A. baumannii*, *P. aeruginosa*, and *Enterobacter* species, responsible for the six main nosocomial infections related to care (contribution 5). However, most new drugs were developed from traditional molecules, and their use may lead to the rapid emergence of resistance. At the same time, ICU patients often experienced altered renal excretion, and various conditions (e.g., obesity, high MICs) may influence the treatment response and potentially determine underdosing, as well as the high effluent rates during CKRT (Table 1).

## 5. Conclusions

In critically ill patients on CKRT, and especially with associated sorbent therapy, the underdosing of antibiotics can become a frequent and potentially more dangerous problem than overdosing. Even considering that TDM is not available worldwide, the risk of nephrotoxicity should not be overestimated. Instead, the patients have to be actively monitored to maintain appropriate drug levels, ensuring their survival and minimizing the risk of therapeutic failure.

## Figures and Tables

**Table 1:** New recently developed antibiotics and potential interference with CKRT. All data included in this table are derived from available official prescribing information and [3].

Agent (Commercial Name)	Antibiotic Class	Potential Nephrotoxicity	Dosing Modification in Renal Impairment	Potential Dose Modification in CKRT
Eravacycline (Xerava^®)^	Tetracycline	No	No	No
Delafloxacin (Baxdela^®)^	Fluoroquinolones	Yes	Yes (not recommended for GFR < 15 mL/min)	Yes
Plazomicin (Zemdri^®^) *	Aminoglycoside	Yes	Yes (insufficient information for GFR < 15 mL/min)	Yes
Sulbactam- durlobactam (Xacduro^®^)	Β-Lactam	Yes	Yes	Yes
Ceftazidime-avibactam (Zavicefta^®^)	Β-Lactam	Yes	Yes	Yes **
Meropenem/vaborbactam (Vaborem^®^)	Β-Lactam	Yes	Yes	Yes
Imipenem/cilastatin/relebactam (Recarbrio^®^)	Β-Lactam	Yes	Yes (do not use unless hemodialysis is instituted within 48 h for GFR < 15 mL/min)	Yes
Ceftolozane/tazobactam (Zerbaxa^®^)	Β-Lactam	Yes	Yes	Yes
Cefiderocol (Fetroja^®^)	Β-Lactam	Yes	Yes	Yes

* Not yet approved in the EU, ** only information on the dosing of Zavicefta^®^ for patients requiring dialysis is in intermittent hemodialysis. For other types of dialysis, it is suggested that the dose/frequency of ceftazidime-avibactam should follow local label/local guidelines for dosing ceftazidime.
